# Gut microbiota and pediatric metabolic dysfunction–associated steatotic liver disease: clinical evidence and therapeutic implications

**DOI:** 10.3389/fped.2026.1773294

**Published:** 2026-05-13

**Authors:** Bhagyalakshmi Nair, Adithya Jayaprakash Kamath, Aswathy R. Devan, Rajesh Gopalakrishna, Ashok R. Unni, Lekshmi R. Nath

**Affiliations:** 1 Department of Pharmacognosy, Amrita School of Pharmacy, Amrita Vishwa Vidyapeetham, AIMS Health Sciences Campus, Kochi, India; 2 Department of Gastroenterology, Amrita Institute of Medical Sciences and Research Centre, Kochi, India; 3 Central Lab Animal Facility, Amrita Institute of Medical Sciences, Kochi, Kerala, India

**Keywords:** gut microbiota, MASH, MASLD, obesity, pediatric population, steatosis

## Abstract

Metabolic dysfunction-associated steatotic liver disease (MASLD), previously referred to as Non-alcoholic fatty liver disease (NAFLD), is among the most common chronic liver conditions globally. The incidence of MASLD has been rising, primarily due to lifestyle changes, excessive calorie intake, and other metabolic conditions like obesity and Type 2 diabetes mellitus in both adults and children. An inclusive understanding of risk factors of childhood MASLD is still unknown. Based on the limited case studies published, familial clustering is prominent in MASLD suggesting role of genetic factors. Moreover, children with MASLD are at higher risk of obesity, diabetes and hypertension. Studies associating the colonic microbiome with MASLD have been limited and complicated by inconsistencies in study design and approach but mostly indicate a role in the pediatric population. The present review provides a comprehensive understanding of the most recent clinical studies (2013–2026) on the role of gut microbiome in the development of pediatric MASLD.

## Introduction

Non-alcoholic fatty liver disease (NAFLD) is a multifaceted condition recognized as a rapidly emerging manifestation of metabolic syndrome (1). The classification of steatosis has evolved from the traditional concept of NAFLD to more inclusive framework of Metabolic dysfunction-associated steatotic liver disease (MASLD). NAFLD is characterised by the presence of liver steatosis that affects around ≥5% of hepatocytes, diagnosed by imaging techniques or histology, in the absence of significant alcohol consumption (2). Its more severe form, Non-alcoholic steatohepatitis (NASH), is characterized by excessive fat accumulation in the liver (steatosis), along with cell damage, inflammation, and hepatocyte death, potentially progressing to fibrosis, cirrhosis, and hepatocellular carcinoma (HCC). In contrast, MASLD features the presence of hepatic steatosis associated with at least one cardiometabolic risk factor, like obesity, type 2 diabetes mellitus, dyslipidemia, or hypertension, thereby correlating metabolic dysfunction into the diagnostic conditions (3).

The progression of NASH to chronic liver disease has now become one of the primary reasons for liver transplantation (4). In 2023, three leading international liver organizations namely, the American Association for the Study of Liver Disease (AASLD), the European Association for the Study of the Liver (EASL), and the Latin American Association for the Study of the Liver (ALEH) suggested replacing the term NAFLD with Metabolic dysfunction-associated steatotic liver disease (MASLD) and renaming NASH as metabolic dysfunction-associated steatohepatitis (2, 5). This updated classification allows to reflect the disease pathogenesis, improve patient-risk stratification, and increase the clinical relevance in therapeutic context.

Recent epidemiological findings demonstrate a strong similarity between the definitions of NAFLD and MASLD, with around 99% of NAFLD cases meet the MASLD criteria. As a result, experts suggests that metabolic dysfunction-associated steatotic liver disease (MASLD) is a more accurate term than NAFLD to represent the current understanding of fatty liver disease (6). Recent evidence highlights metabolic dysfunction–associated steatotic liver disease (MASLD/NAFLD) as an increasingly significant global health concern in the pediatric population. Meta-analyses involving millions of children worldwide estimate an overall prevalence of approximately 14% in the general pediatric population, rising sharply to around 38% among obese children. Other population-based studies report prevalence rates ranging from 3% to 10% in unselected pediatric groups, with MASLD affecting nearly one-third of obese boys and one-quarter of obese girls (7).Regionally, some of the highest rates are documented in North Africa and the Middle East, where adolescent MASLD prevalence is rapidly increasing. Global projections are equally concerning: the total burden of MASLD is expected to reach nearly 460 million cases by 2030, with further increase to over 520 million cases by 2050. Among children and adolescents with overweight or obesity, prevalence surpasses 40%, underscoring the urgency for early identification, effective prevention programs, and targeted therapeutic interventions (8).

The risk of MASLD is directly correlated with the Body Mass Index (BMI) suggesting that obese children are at the highest risk (9). Although obesity is a key risk factor, MASLD can also develop in individuals who are not obese. This group is often referred to as “lean NAFLD” or “non-obese NAFLD” (10). Ethnicity and genetic factors could be modulating factors other than insulin resistance especially in lean NASH patients (11). Studies suggest that MASLD in children may have its origins *in utero* for obese, insulin-resistant mothers (12). A study used neo-natal magnetic resonance spectroscopy to quantify steatosis in infants born to moms with gestational diabetes. The mean hepatic fat fraction of the newborns born to obese mothers with gestational diabetes was 68% higher than that of the infants born to mothers of normal weight (13). Diet is also a major factor which influences the gut microbial composition. On starting the intake of solid food, the gut microbiota expands to its diverse composition (14). Compared the gut microbiota in children 6–10 years of age of two different dietary habits. Compared to the European pediatric population, the African pediatric population had a healthy gut microbial composition which is due to their high fibre consumption than European population. It was also found that the African population had a predominance of *Prevotella* species (14). This review highlights the current understanding of the role gut microbiota plays in the progression of metabolic-associated fatty liver disease (MASLD), with a particular focus on its impact on the pediatric population (aged form birth to 18 years) based on the clinical evidence.

## Methodology

**Figure 1:**
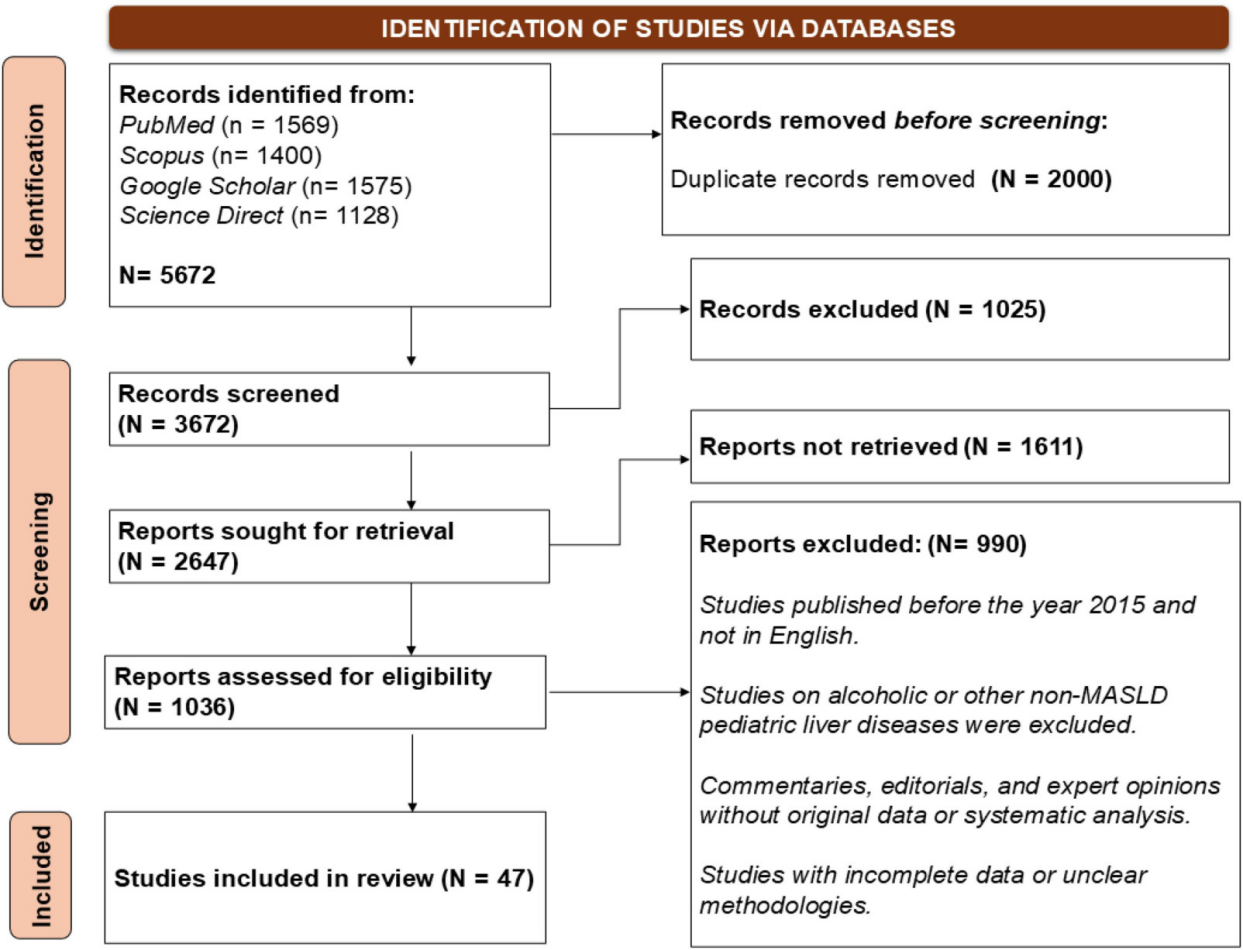
The above figure represents the PRISMA *(preferred reporting items for systematic reviews and meta-analyses)* flowchart of studies selected using various electronic databases like pubMed/medLine, scopus, google scholar, and science direct. The review includes a total **47** articles after the screening and exclusion.

A structured literature search was conducted to identify studies published between January 2013 and April 2026. Databases included *PubMed/MEDLINE, Scopus, ScienceDirect, and Google Scholar*. Priority was given to clinical studies involving pediatric populations, including observational studies, randomized controlled trials, systematic reviews, and meta-analyses. Selected preclinical studies were considered where necessary to support mechanistic interpretation. Both Medical Subject Headings (MeSH) and free-text terms were used. Core search combinations included “*gut microbiota,” “microbiome,” or “intestinal microbiome” with “NAFLD,” “NASH,” “MASLD,” “MASH,” or “fatty liver,” and “children,” “pediatric,” or “adolescent,”* using Boolean operators. Because most earlier studies used NAFLD/NASH terminology, these terms were included in the search. For clarity, findings are discussed using MASLD/MASH nomenclature while retaining the original terminology where relevant. A total of 47 articles met the overall inclusion framework. 11 provided primary pediatric clinical data (6 observational studies and 5 interventional trials). Study selection involved screening of titles and abstracts followed by full-text review against predefined criteria (Figure 1).

### Inclusion criteria

- English-language publications
- Studies evaluating gut microbiota in relation to MASLD pathogenesis, progression, or treatment
- Pediatric population (birth–18 years)
- Clinical studies [Randomized controlled trials (RCTs), cohort, case–control], systematic reviews, meta-analyses, and relevant mechanistic studies

### Exclusion criteria

- Publications prior to 2013
- Non-English articles
- Studies focused on alcoholic liver disease or unrelated hepatic conditions
- Editorials or commentaries without primary data
- Studies with incomplete methodology or unclear outcomes

For each study, data were extracted on design, sample size, age range, diagnostic criteria, microbiome assessment methods, intervention details, and principal clinical and microbial findings. Given the small number of pediatric studies and their heterogeneity, formal risk-of-bias scoring was not undertaken. Instead, studies were appraised qualitatively based on cohort size, diagnostic rigor, control of confounding factors such as obesity and diet, microbiome methodology, and duration of follow-up.

### Role of gut microbiota in regulating homeostasis in healthy individuals

The gut microbiota is a key regulator of human health, contributing essential metabolic, immune, and endocrine functions that support homeostasis. As a metabolically active ecosystem, it facilitates the digestion of complex dietary components and produces a range of bioactive metabolites, including short-chain fatty acids (SCFAs), which modulate inflammatory responses, maintain gut barrier function, and influence host energy metabolism. Through these metabolic activities, the microbiota shapes nutrient absorption, regulates gut hormone secretion, and plays a central role in glucose homeostasis and energy balance (15). Beyond its metabolic contributions, the gut microbiota supports mucosal integrity and immune balance by interacting directly with epithelial and immune cells. These interactions strengthen barrier defense, promote immune tolerance, and provide colonization resistance against pathogenic organisms. The microbiota also influences metabolic pathways, contributing to the regulation of oxidative stress, energy balance, and inflammatory tone. When this finely tuned ecosystem is disrupted—a state known as dysbiosis—it can impair host metabolic and immune functions and has been strongly associated with a range of chronic conditions, including obesity, diabetes, inflammatory bowel disease, and non-alcoholic fatty liver disease (16).

The gut microbiota generates a range of bioactive compounds—including short-chain fatty acids (SCFAs), neurotransmitters, and other signaling molecules—that either diffuse across the intestinal barrier or engage specific receptors on host cells. These metabolites modulate gene expression within intestinal epithelial cells and regulate the secretion of key enteroendocrine hormones such as glucagon-like peptide-1 (GLP-1) and peptide YY (PYY), which are central to appetite regulation, glucose homeostasis, and overall energy balance. In this way, microbial metabolic activity is directly linked to host metabolic control (17). Overall, the gut microbiota functions as a dynamic metabolic organ intricately integrated with host biology. By maintaining metabolic homeostasis, supporting immune equilibrium, and protecting against pathogens, it plays a fundamental role in health and contributes to disease prevention across multiple organ systems (18).

### The developing gut ecosystem: distinct microbial signatures and its relevance in children

The pediatric gut microbiota represents a rapidly evolving ecosystem marked by distinct microbial signatures that undergo substantial shifts during early life. In infancy, the gut community is relatively simple and dominated by *Bifidobacteriaceae*, which play a central role in breaking down human milk oligosaccharides. As complementary foods are introduced around six months of age, microbial diversity increases, accompanied by a rise in taxa such as *Bacteroidetes* and *Firmicutes*, gradually steering the microbiome toward a more adult-like composition. This transition enhances metabolic capacities, including carbohydrate fermentation and vitamin synthesis, which are essential for supporting growth and developmental needs (19). Microbial diversity continues to expand through early childhood, typically stabilizing between three and five years of age. The gut microbiota remains dynamic and responsive throughout later childhood and adolescence, with factors such as diet, environmental exposures, antibiotic use, and lifestyle influencing its composition and function. This early developmental window is critical, as microbial interactions during this period shape immune maturation, epithelial barrier function, and long-term metabolic programming, thereby influencing susceptibility to chronic diseases later in life (20).

**Figure 2:**
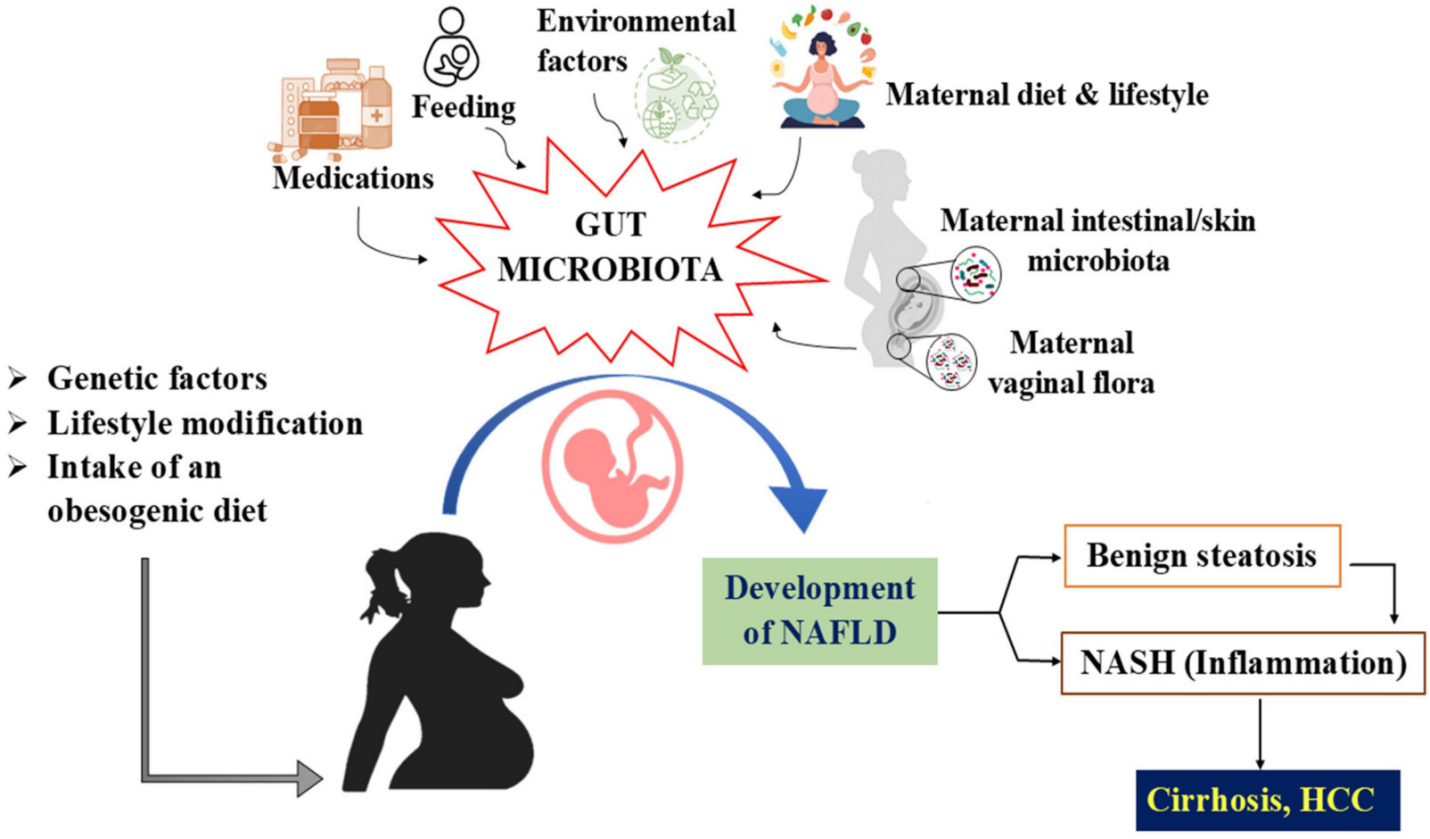
Represents the maternal and early-lifestyle factors of gut microbiome and their role in the development of pediatric MASLD. Maternal factors such as diet, lifestyle modifications, environmental exposures, medications, feeding practices, and maternal intestinal/skin and vaginal microbiota influence the development of infant gut microbiome. In addition, other factors like genetic predisposition, and intake of an obesogenic diet, early microbial alterations may contribute to the development of MASLD *in utero* and early life.

Pediatric microbial population exhibit several features that differentiate them from adult population. Children generally display lower overall microbial diversity and higher relative abundances of gram-negative taxa, including *Bacteroidia* and certain *Proteobacteria*, as well as elevated levels of *Bacteroides*. In contrast, adults harbour a more complex and diverse microbiota enriched with Firmicutes such as *Blautia*. Functional distinctions parallel these compositional differences: pediatric microbiomes tend to support glycan degradation pathways and the biosynthesis of vitamins such as riboflavin (B2), pyridoxine (B6), and folate (B9), along with enhanced amino acid catabolism. With maturation, the microbiota shifts toward increased carbohydrate metabolic pathways and the biosynthesis of thiamine (B1), pantothenic acid (B5), and other metabolites characteristic of adult microbial communities (21, 22). Importantly, the developing gut microbiota in pediatric population is more susceptible to dysbiosis than the adult microbiome. Early exposures such as caesarean delivery, formula feeding, reduced dietary fiber intake, and antibiotic administration can alter the trajectory of microbial maturation, potentially increasing the risk of allergic diseases, obesity, and inflammatory conditions. The responsiveness of the pediatric microbiota to dietary and metabolic factors also has implications for conditions such as pediatric MASLD, where altered microbial composition and metabolism may influence disease onset and progression (23). Therefore, the gut microbiome in children progresses from a simple neonatal community to a complex, stable, adult-like ecosystem during early childhood. This maturation is fundamental to immune development, metabolic balance, and lifelong disease risk, underscoring the importance of early-life microbial patterns in shaping long-term health outcomes (Figure 2).

Growing evidence indicates that alterations in the developing gut microbiome play a pivotal role in the onset and progression of pediatric MASLD, influencing metabolic regulation, immune maturation, and hepatic inflammation. The relationship between the gut microbiome and MASLD-related liver disorders has therefore gained considerable attention, with gut dysbiosis now recognized as a key disruptor of gut–liver homeostasis and a major contributor to disease onset and progression (24). In children with obesity and MASLD, the gut microbiota composition shows distinct alterations compared with healthy children. Childhood obesity is often associated with an increased *Firmicutes-to-Bacteroidetes* ratio, which may enhance energy harvest from the diet and contribute to excessive weight gain. Obese children also tend to exhibit elevated levels of *Streptococcus, Acidaminococcus*, and *Prevotella*, along with a reduction in beneficial butyrate-producing microbes essential for maintaining gut barrier integrity and exerting anti-inflammatory effects (25). In the context of MASLD, dysbiosis is further characterized by an increased abundance of pro-inflammatory and ethanol-producing bacteria, alongside decreased overall microbial diversity. This dysbiotic state promotes increased intestinal permeability, enabling translocation of bacterial endotoxins to the liver, where they trigger inflammation and lipid accumulation, critical factors of MASLD progression in pediatric patients (26).

Altered microbial fermentation activity in obese children—including elevated production of short-chain fatty acids (SCFAs) such as acetate, propionate, and butyrate—adds another layer of metabolic disturbance. While butyrate typically supports gut health, excess acetate and propionate may contribute to hepatic lipogenesis and insulin resistance (27). Overall, children with obesity and MASLD exhibit gut microbiota signatures marked by dysbiosis, an increased *Firmicutes-to-Bacteroidetes* ratio, depletion of beneficial bacteria, and altered microbial metabolites linked to inflammation and metabolic dysfunction. These changes not only fuel obesity and fatty liver disease but also represent potential targets for microbiome-based therapeutic interventions. Microbial colonization begins at birth—and possibly during pregnancy—with maternal vaginal, gut, and skin microbiota serving as initial sources of inoculation (13). During infancy, the gut microbiome is dominated by *Bacteroides* (phylum Bacteroidetes), *Lactobacillus* (phylum Firmicutes), and *Bifidobacterium* (phylum Actinobacteria), with *Proteobacteria* also abundant in breastfed infants (28).

**Figure 3:**
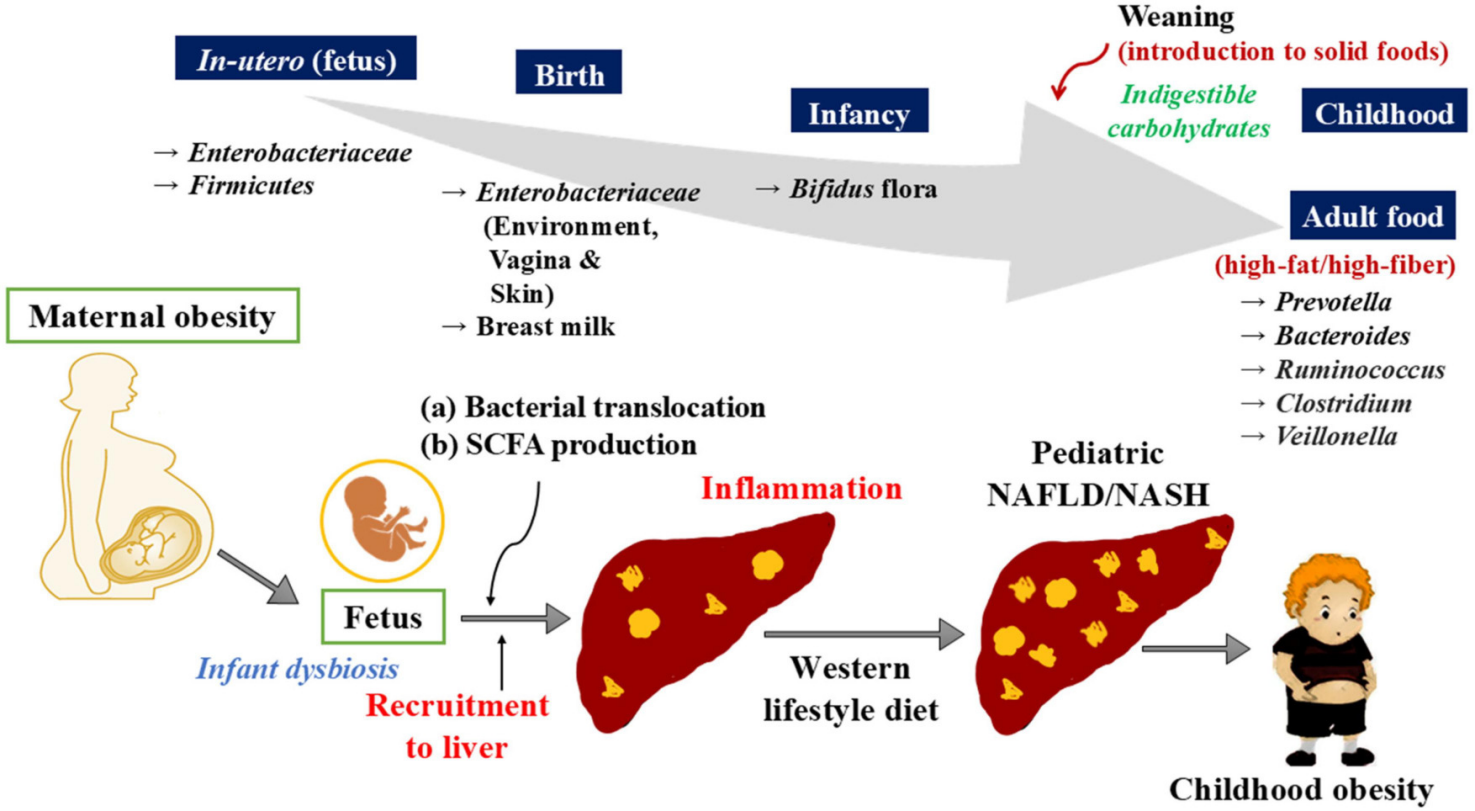
Represents the gut microbial composition in different stages of childhood development. The establishment of the gut microbiota begin *in utero* and dominated by *Enterobacteriaceae* family, and *Firmicutes* phylum. After birth, a newborn's gut is initially dominated by *Enterobacteriaceae*, followed by a shift to *Bifidobacterium* and lactic acid bacteria, known as “Bifidus flora.” This persists until solid foods are introduced. With weaning, adult-type bacteria like *Bacteroides*, *Prevotella*, *Veillonella* and *Ruminococcus* colonize the gut, and by age three, the child's microbiota resembles that of an adult. (b) Maternal obesity can lead to dysbiosis in the infant, characterized by increased short-chain fatty acid production and a leaky gut, allowing bacterial translocation. In the liver, this translocation triggers inflammation, and when combined with exposure to high-calorie foods, such as a Western-style diet, it contributes to the development of hepatic steatosis and obesity.

Lim et al. reported that healthy infant twins displayed notable levels of *Clostridia, Bacilli, Gammaproteobacteria,* and *Actinobacteria* and demonstrated that the absence of *Bifidobacteria* can facilitate pathogenic colonization and disease progression (29). Consistent with this, Yan et al. showed that supplementation with *Bifidobacterium animalis* subsp. *lactis* V9 ameliorated MASLD in rats by reducing expression of sterol regulatory element-binding protein 1c (SREBP1c) and fatty acid synthase (FAS), decreasing hepatic PPAR-α mRNA levels, and activating the AMPK pathway. Additionally, co-administration of *Bifidobacterium* with dietary blueberries has been shown to reduce hepatic lipid accumulation in rats (30). These findings highlight the importance of *Bifidobacteria* during infancy (up to 2 years), suggesting that early-life microbiome composition may directly or indirectly influence MASLD risk in children. Between 18 and 36 months, the infant gut microbiome undergoes a critical developmental transition, becoming more stable and increasingly dominated by phyla *Bacteroidetes* and *Firmicutes* (31). This shift is strongly influenced by the introduction and diversification of solid foods. Several studies have demonstrated that microbial complexity increases markedly following the introduction of solids (32), and that earlier dietary diversification accelerates the transition toward an adult-like microbial profile. In adults, the gut microbiome typically comprises six to seven dominant phyla, with *Bacteroidetes* and *Firmicutes* being predominant, whereas *Proteobacteria*, *Verrucomicrobiota*, *Actinobacteria*, and *Euryarchaeota* are present in smaller proportions (33), (Figure 3).

### Implications of the gut microbial-liver relations in pediatric population

#### Clinical evidence

A clinical study reported by *Del Chierico* et al. (34) investigated the relationship between gut microbiota and the liver using the “*omics”* approach. In this study, children in the age group of 7–16 years of age were the subjects. A total of 61 patients [27 MASLD (formerly NAFLD) patients, 26 MASH (formerly NASH) patients, and eight obese patients] were compared to 54 control subjects of the same age group. As per the study outcome, an increase in *Ruminococcus* was identified as a microbiota mark of MASLD onset, and an increase in *Dorea* genus was identified as a microbiota sign of the MASLD-MASH progression stage. *Oscillospira,* a gut bacteria involved in the production of butyrate, may impair gut barrier integrity, elevating microbial products (e.g., LPS) and metabolites like 2-butanone via portal circulation, that leads to hepatic inflammation, steatosis, and fibrosis through TLR signaling and activating the Kupffer cells and stellate cells. However, Inconsistencies in taxa (e.g., *Oscillospira* depletion vs. variable *Prevotella* abundance) are the results of heterogeneous study designs, small sample size, regional and dietary variables, as well as the limits of 16S sequencing and strain-level functional variations (34).

*Zhu* et al. (35) reported a clinical study that investigated the profile of the gut microbiota in MASH (formerly NASH) patients of the pediatric population. Three groups of children, which consisted of 16 control, 25 obese patients, and 22 MASH patients of the age group of 12–16 years, were selected as the subjects. On assessing their bacterial population of gut microbiota, it was revealed that each group had a unique gut microbiota profile. There were notable differences between the groups at various levels of bacterial classification (genus, family, and phyla levels). The *Proteobacteria*, *Enterobacteriaceae* and *Escherichia coli* are the only group of phylum, family, and genus types that exhibited significant difference in the obese and MASH groups. The concentration of blood alcohol producing bacteria was similar in healthy cohorts and obese non-MASH patients, but the MASH patients exhibited prominently higher levels of blood ethanol levels. The study observed an elevated levels of alcohol-producing bacteria in the gut microbiome of MASH patients. The elevated levels of blood alcohol concentrations observed in MASH subjects suggest increased endogenous alcohol production, driven by ethanol-producing *Escherichia* species, which may contribute to the progression from obesity to MASH (35).

Another study published in 2019 shows the effect of intestinal microbiota on the progression of MASLD (formerly NAFLD). The study subjects included pediatric patients with and without MASLD aged 8–17 years. Ecological diversities were seen differently in children with MASLD than in the other group. In the case of children with MASH, genes that regulate lipopolysaccharide synthesis that encodes for 3-deoxy-D-manno-octulosonate 8-phosphate-phosphatase were found to be enriched in their gut microbiomes. High levels of *Prevotella copri* was associated with lower α-diversity and moderate-to-severe fibrosis. The study depicts that pediatric MASLD is associated with two-community level alterations in the gut microbiome. First, there is a significant decrease in the α-diversity, indicating diminished microbial richness and abundance. Secondly, there is an increased inter-individual variability, suggesting greater person-to-person differences in gut microbiome composition. High levels of *Prevotella copri* and *Proteobacteria* leads to dysbiosis and transfer of bacterial endotoxins, which cause hepatic inflammation and Toll-like receptor signaling. In contrary, reduced *Firmicutes* and taxa like *Akkermansia* impair SFAs production and bile acid metabolism, exacerbating steatosis and fibrosis through altered energy harvest and immune modulation. In children, these microbial alterations increase the person-to-person variability and promote inflammation that contributes to liver injury, even when the overall metagenomic gene abundance remains unchanged (36).

An EPOCH (Exploring Perinatal Outcomes among Children) study investigated the connection between gut microbiota and hepatic fat fraction (HFF) among 107 children 12–19 years of age. On examining the faecal samples using 16S rRNA sequencing, higher hepatic fat fraction variants had low alpha manifold, and the dietary intake of these variants was defined with a high intake of monounsaturated fatty acids. The study successfully established a strong connection between gut microbiota and HFF. The dysbiosis disrupts the intestinal barrier and enables the portal translocation of LPS (lipopolysaccharide). Also, metabolites like ethanol promote the TLR4-mediated inflammation and TNF-α production. SCFAs lowers the level of butyrate/acetate impairing intestinal integrity, insulin sensitivity, and hepatic lipid metabolism via GPR43/FXR pathways. The altered bile acid profiles from taxa like *Bilophila* (taurine-conjugated bile tolerant) further dysregulate hepatic lipogenesis and gluconeogenesis. The study does not establish a definitive conclusion regarding the progression of the association between gut microbiota and liver steatosis, as it remains unclear whether the emphasized microbial taxa contribute causally to fatty liver development or simply reflect underlying obesity and its metabolic consequences (37).

A clinical study published in 2015 evaluated the gut microbial profiles of three groups of obese, lean and healthy children with MASLD (formerly NAFLD) and without MASLD. On analysing the faecal specimens through 16S rRNA Gene microarray, meta proteomics, metagenomics and shotgun sequencing, it was evident that the pediatric population with MASLD shows presence of *Gamma proteobacteria* and *Prevotella*. Moreover, the children with MASLD expressed a significant increase in microbiome population responsible for SCFA synthesis and ethanol production that weaken the gut barrier integrity. Further, validation studies are essential to guide future microbiome-targeted interventions intended at correcting the dysregulated metabolite profile associated with MASLD (38). A double-blind, randomized controlled trial (RCT) was conducted in 44 obese children diagnosed with biopsy-confirmed MASLD. The study evaluated the effects of probiotic formulation containing eight strains: *Streptococcus thermophilus, bifidobacteria (B. breve, B. infantis, B. longum),* and *Lactobacillus species (L. acidophilus, L. plantarum, L. paracasei,* and *L. delbrueckii subsp. bulgaricus*), over four months. Results showed that the probiotic mixture significantly improved fatty liver disease, primarily by increasing serum GLP-1 levels. The dual beneficial effects of this probiotic formulation was observed due to its direct or indirect effect of induced gut microbiome balance. These probiotics significantly increased the GLP-1 expression and improved the insulin sensitivity and reduced the metabolic stress. Although the study shows that this probiotic supplementation reduces hepatic steatosis and improves BMI in children with NAFLD, the long-term effects of its effect on reversing hepatic injury in MASH, along with comprehensive metabolomic and gut microbiota analyses, remain to be clarified in future extended trials (39).

The probiotic formulation restores microbial balance and reinforces intestinal barrier integrity, thereby reducing gut permeability and limiting lipopolysaccharide (LPS) translocation to the liver—a key trigger of hepatic inflammation and progression of MASLD. By decreasing endotoxin burden, the probiotic directly attenuates activation of hepatic inflammatory pathways and reduces the “second hit” of oxidative stress and cytokine release that drives hepatocellular injury. In addition, the probiotic modulates bile acid composition and influences farnesoid X receptor (FXR) and TGR5 signaling, mechanisms known to be disrupted during dysbiosis. Normalization of these pathways supports improved lipid oxidation, reduced hepatic lipogenesis, and attenuation of inflammatory signaling within the liver. These effects align with the observed clinical reduction in liver fat content and inflammatory markers (40). Furthermore, it enhances hepatic expression of peroxisome proliferator-activated receptors (PPARs), which regulate fatty acid oxidation and exert anti-fibrotic effects. Evidence from animal models shows that the probiotic mixture decreases collagen accumulation and inhibits hepatic stellate cell activation—central drivers of fibrosis—through modulation of TGF-β and matrix metalloproteinases (MMPs). This mechanistic profile supports the improved fibrosis in MASLD patients and underscores the relevance of microbiome modulation in slowing disease progression (41). Importantly, the rise in GLP-1 levels observed in the RCT provides a direct metabolic link between gut microbial restoration and hepatic improvement. GLP-1 enhances insulin sensitivity, suppresses hepatic *de novo lipogenesis*, and exerts anti-inflammatory effects, collectively reducing steatosis and improving overall liver metabolic health in children with MASLD (42).

*Ji* et al. (43) recently conducted a case-control study involving 52 children aged 5–11 years with newly diagnosed MASLD and 52 healthy children to explore changes in the gut microbiome associated with MASLD. Stool samples were collected for 16S rRNA gene sequencing, and the Shannon and Chao indices were used to assess gut microbiota diversity. Principal coordinates analysis (PCoA) was performed to evaluate beta diversity between the two groups. The study revealed that children with MASLD had a significantly different gut microbiota composition and lower alpha diversity compared to the control group, indicating reduced microbial richness and diversity. The children with MASLD exhibited a decreased relative abundance of the phyla *Verrucomicrobia* and *Desulfobacterial,* and the genera *Blautia, Lachnospiraceae NK4A136 group, Coprococcus, Erysipelotrichaceae_UCG-003, UCG-002,* and *Akkermansia*. Additionally, the species *Bifidobacterium longum* was less abundant in children with MASLD. The study also found that the ratio of *Bacteroidetes to Firmicutes (B:F)* (*P* = 0.002) was significantly higher in children with MASLD compared to the healthy group. The study observed a prominent decrease in the relative abundance of *Blautia* genus and *Coprococcus* in MASLD children. *Blautia* and *Coprococcus* regulates short-chain fatty acids (SCFAs) production, the primary metabolites generated through dietary fiber fermentation in the gut—mainly *acetate*, *propionate*, and *butyrate*. These SCFAs serve as key energy sources for intestinal epithelial cells and are integral to immune modulation, lipid and glucose metabolism, and the maintenance of microbial homeostasis. Disruption of their production may impair gut barrier integrity and promote hepatic lipid deposition. These findings suggest that notable alterations in the gut microbiota are present in children with MASLD, which may contribute to the development of the disease (43).

In a double-blind placebo-control RCT by *Nicolucci* et al. children aged 7–12 years with overweight or obesity (>85th percentile of body mass index) received prebiotics comprising oligofructose-enriched inulin and maltodextrin placebo for 16 weeks. Results indicate that prebiotics considerably lowers body weight and fat percentage. The sequencing analysis shows selective gut microbial changes including, significant increase in the OTUs (Operational Taxonomic Unit) of *Bifidobacterium longum* in the MASLD patients (*after receiving the prebiotic supplementation*), a decrease in the *Clostridium clostridioforme,* a pathogenic bacteria associated with serious and invasive human infection. In the prebiotic group, a decrease in the *Bacteroides vulgatus*, positively correlated with normalized weight gain and reduced the trunk body fat. A significant *Bifidogenic* response was achieved with a relatively low dose (8 g/day), compared with higher doses typically required in adults (44). A randomized triple-masked controlled trial comprised 56 pediatric subjects with BMI greater than or equal to 85%. The patients received Synbiotic capsules [Protexin, 2.0 × 10^8^ colony-forming units (CFU)/day] for 8 weeks. The capsule is a mixture of viable and frozen dried *Lactobacillus. casei, Lactobacillus. rhamnosus, Streptococcus. thermophilus, Bifidobacterium. breve, Lactobacillus. acidophilus, Bifidobacterium. longum, Lactobacillus. bulgaricus* of human origin with prebiotics (frocto oligosaccharides), Vitamin E, Vitamin A, and Vitamin C. The Synbiotic capsule supplementation significantly decreased the TNF-a and IL-6 concentration with increase in the adiponectin levels. Synbiotic supplementation may possibly exert its effect by modulating the gut microbiome composition and enhance the growth of beneficial bacteria such as *Lactobacillus and* elevate the SCFA production by improving the *Firmicutes/Bacteroidetes* ratio commonly observed in obesity. Subsequently, synbiotic intake attenuate the inflammatory pathways by increasing the level of anti-inflammatory adiponectin levels and reduced pro-inflammatory cytokines, including TNF-α and IL-6 (45).

In a triple-blind randomized controlled trial (RCT), 64 obese pediatric patients were given either a probiotic capsule (constituting 3 × 10⁹ CFU of *Lactobacillus acidophilus* ATCC B3208, 6 × 10⁹ CFU of *Bifidobacterium lactis* DSMZ 32269, 2 × 10⁹ CFU of *Bifidobacterium bifidum* ATCC SD6576, and 2 × 10⁹ CFU of *Lactobacillus rhamnosus* DSMZ 21690) or a placebo for 12 weeks. The results showed that probiotic supplementation led to decreased serum AST, ALT, Cholesterol, LDL-C, Triglycerides, and waist circumference. The liver ultrasonography showed that Synbiotic supplementation decreased the prevalence of high-grade fatty liver in intervention group compared to placebo. The probiotic supplementation restores the microbial diversity associated with the lower gut-derived LPS endotoxemia and lowers the *Proteobacteria* abundance. This decreases TLR4 activation, thereby attenuating inflammation and hepatic fat accumulation. Additionally, probiotic supplementation recovers intestinal integrity and limits bacterial translocation. However, there was no significant effect on BMI or BMI z-score (46). Another study by Du et al. reported changes in the microbiome signatures and microbial metabolites of children with MASLD. The study employed children and adolescents from 6 to 16 years (*n* = 36 for 16S rDNA sequencing and *n* = 25 for targeted metabolomics). The results showed significantly lower *α*-diversity in children with obesity and MASLD compared with healthy controls. Further analysis identified *Anaerostipes hadrus* as the top biomarkers differentiating the obesity group from the MASLD group. The study highlights increased abundance of *Ruminococcus torques (R. torques)*, which was positively correlated with the levels of deoxycholic acid (DCA). Importantly, a positive correlation was observed between fecal levels of *R. torques* and DCA, indicating that DCA could be a product of *R. torques* (47). A case-controlled study by Rohani et al. investigated the link between gut microbiome composition and MASLD in overweight and obese Iranian children (*n* = 66). The study observed lower abundance of *Coprococcus* bacteria in the MASLD group compared to healthy control. Additionally, there was an increase in the pro-inflammatory bacteria, *Prevotella.* The *Coprococcus* species showed negative correlation with ALT levels, suggesting the protective role for *Coprococcus* genera. The *Prevotella* genus depicted significant positive correlation with both ALT and AST levels, implying its role in liver-related injury. Further validation using long-term follow-up studies using integrated metabolomic and immune profiling are required to provide deeper insights into gut microbiome and MASLD interactions (24) (Table 1).

**Table 1:** Tabular column representing the clinical trials of pediatric population associated with MASLD.

Sl. no	Study design	Study population	Age of the population	Study type	Study results	Reference/Clinical Trial No.
1	Case control observational study	61 patients (27 NAFL patients, 26 NASH patients, and 8 obese patients)	7–16 years	Metagenomics and Metabolomics	Increase in *Ruminococcus* as a microbiota mark of MAFL onset. Increase in *Dorea,* as a microbiota sign of the MASLD/MASH progression stage.	Del Chierico et al., (34)
2	Case control Observational study	25 obese patients, 16 control, and 22 NASH patients	12 to 16 years	16S ribosomal RNA (rRNA) pyrosequencing	Elevated levels of ethanol producing bacteria (*Escherichia*) in MASH subjects. *Proteobacteria*, *E.coli*, and *Enterobacteriaceae* levels are more in MASH patients.	Zhu et al., (35)
3	Prospective, Observational, Cross-sectional study	87 children (with biopsy-proven NAFLD) & 37 children (controls)	8–17 years	16S ribosomal RNA amplicon sequencing.	Children with MASLD/MASH had lower gut microbiome diversity compared to healthy controls. Increased levels of *Prevotella copri*	Schwimmer et al., (36)
				Metagenomic shotgun sequencing		
4	Prospective-cohort study (EPOCH study)	107 children	12–19 years	16S rRNA gene sequencing	Lower alpha diversity was associated with higher hepatic fat fraction (HFF).	Stanislawski et al., (37)
5	Cross-sectional, Case control, Observational study	26 lean healthy controls, 13 obese children without NAFLD, & 11 obese children diagnosed with NAFLD	13 years	16S rRNA gene microarray	Pediatric population with MASLD had *Gamma proteobacteria* and *Prevotella*. Children had excessive levels of ethanol with short chain fatty acids.	Michail et al., (38)
				High-throughput DNA sequencing Proteomics		
6	Case-control study	104 subjects	5–11 years	16S rRNA gene sequencing	Children with MASLD exhibited a decreased relative abundance of the phyla Verrucomicrobia and Desulfobacterial, the genera Blautia, Lachnospiraceae_NK4A136_group, Coprococcus, Erysipelotrichaceae_UCG-003, UCG-002, and Akkermansia. Ratio of Bacteroidetes to Firmicutes (B:F) was significantly higher in children with MASLD compared to the healthy group	Ji et al., (43)
				Shannon and Chao indices to evaluate the gut microbial diversity		
		52 MASLD children & 52 healthy children				
				Principal coordinates analysis (PCoA)		
7	Case-controlled study	66 children	Mean age of the study population is 136.82 month	Anthropometry	Reduced abundance of *Coprococcus* genus and increased pro-inflammatory bacteria (e.g., *Prevotella*). A negative correlation existed between *Coprococcus* abundance and ALT levels. *Prevotella* showed positive correlations with both ALT and AST, indicating potential liver injury.	Rohani et al., (24)
				Genomic DNA extraction		
				Microbiome composition analysis		
8	Double-blind, randomized controlled trial	44 obese children with biopsy-proven MASLD	9–12 years	Anthropometry	Improved fatty liver disease and BMI Increased serum GLP-1 levels	NCT01650025; Alisi et al., (39)
				Blood test analysis		
				GLP-1 Assay		
				Liver Ultrasonography		
9	Single-center, Double-blind, Placebo-controlled trial	22 children in OI (oligofructose-enriched inulin) group	7–12 years	Dual energy x-ray Absorptiometry.	Reduced the body weight and fat percentage. Increased the *Bifidobacterium* species. Decreased serum triglycerides	NCT02125955; Nicolucci et al., (44)
				Lipid analysis		
				16S rRNA sequencing		
		20 children in Placebo group		Quantitative Polymerase Chain Reaction (q-PCR)		
10	Randomized triple-masked controlled trial	56 pediatric subjects	6–18 years	Biochemical analysis	Decreased the TNF-a and IL-6 concentration with increase in the adiponectin levels	IRCT201103081434N4; Kelishadi et al., (45)
				Bacterial count determination in stool samples		
11	Open-label randomized controlled trial	77 Obese children & 40 Control group	5–17 years	Obesity related anthropometric measurements	Decreased the serum total cholesterol, LDL-C, and total oxidative stress levels. Reduced BMI, and body weight.	NCT01927107
				Evaluation of biochemical indices and oxidative stress		
12	Randomized Triple-blind Trial	64 obese pediatric patients	12 years	Liver function analysis	Liver ultrasonography revealed reduced high-grade fatty liver in MASLD subjects Reduced the serum AST, ALT, cholesterol, LDL-C, triglycerides, and waist circumference. No significant effect on BMI or BMI z-score	IRCT2013100414882N1; Kelishadi et al., (45)
				Lipid profile analysis		
				Anthropometric measurements		
				Liver Ultrasonography		

## Conclusion and future perspective

Pediatric MASLD represents the growing global health concern, with gut microbiome emerging as a central modulator of disease onset and progression. In children, early-life factors such as mode of delivery (vaginal vs. C-section), breastfeeding practices, and dietary patterns play a crucial role in modulating the gut microbiome during the development, thereby impacting metabolic balance and liver health. While pediatric MASLD shares many similarities with the adult form, there are notable differences in terms of prevalence, histology, diagnosis, and management. These differences include the influence of early-life factors, unique risk profiles, variations in diagnostic approaches and treatment responses, and the presence of other underlying conditions, such as inherited metabolic disorders that may require specific interventions. Collectively, the current literature review analysis supports a mechanistic link between the gut dysbiosis, impaired intestinal integrity, and altered bile acid metabolism among the pediatric patients with MASLD. Lifestyle modifications remains the preliminary approach of management with more emphasis on balanced dietary patterns rich in fiber, polyphenols, and unsaturated fats, alongside regular physical activity to improve insulin sensitivity and hepatic fat accumulation. Emerging microbiota-directed therapies including probiotics, prebiotics, synbiotics, postbiotics, and dietary fiber supplementation demonstrate promising potential in restoring microbial eubiosis and attenuating hepatic inflammation. Additionally, modulation of bile acid signaling and microbial metabolites represents a novel mechanistic avenue for therapeutic development.

Probiotic supplementation particularly multi-strain formulations containing *Lactobacillus*, *Bifidobacterium*, and *Streptococcus* species—has demonstrated potential benefits by improving hepatic steatosis, enhancing gut barrier function, reducing endotoxin translocation, and elevating GLP-1 levels, thereby improving insulin sensitivity and lipid metabolism. Beyond metabolic effects, probiotics exert anti-inflammatory actions by suppressing cytokines such as TNF-α and IL-8 and may attenuate fibrosis through modulation of TGF-β signaling and matrix metalloproteinases, as shown in preclinical models. Synbiotics and prebiotics provide additional avenues by supporting the survival and activity of beneficial microbes, although pediatric data remain limited. Dietary modification continues to be the primary approach to management, while microbiome-targeted strategies can serve as valuable adjuncts. These approaches may help optimize bile acid signaling via FXR and TGR5 pathways, restore a balanced *Firmicutes-to-Bacteroidetes* ratio, and decrease the production of harmful metabolites such as LPS and trimethylamine N-oxide (TMAO). More advanced interventions including fecal microbiota transplantation (FMT) and multi-omics–driven personalized therapies integrating microbiome, metabolome, and host genetics are being explored and may ultimately enable individualized treatment options for children with refractory or progressive disease.

The current clinical evidence linking gut microbiome and pediatric MASLD are hindered by limited sample size and short-follow up durations which compromises the strength and generalizability of the study findings. Studies suggest that microbiome-targeted interventions may influence disease progression and its associated metabolic outcomes. The heterogenicity in study design, population characteristics, and outcome measures further delays the disease identification. This present review provides a comprehensive and updated synthesis of current clinical evidence on the role of the gut microbiome in pediatric MASLD. It uniquely emphasizes pediatric-specific microbial signatures, early-life influences, and mechanistic pathways linking gut dysbiosis to hepatic inflammation, and metabolic dysfunction. The manuscript also incorporates the findings from probiotic, prebiotic, and synbiotic interventional trials to highlight translational potential. The pediatric evidence base remains limited. Most studies involve small cohorts, heterogeneous populations, variable methodologies, and short follow-up. Histology-based outcomes are rare, and longitudinal data are scarce. Causal relationships cannot yet be established. In addition, differences in probiotic strains, dosages, and external factors such as diet, antibiotic use, and ethnicity make it difficult to develop standardized therapeutic strategies. Consequently, the field is still evolving, and there is a clear need for comprehensive, thoroughly standardized, and long-term investigations to validate these relations and identify microbiome-based therapies as reliable and established clinical methods in pediatric MASLD.

Future clinical studies focusing on mother–child pairs, gut microbiota composition, and liver health during gestation, infancy, and childhood may help clarify the factors contributing to gut dysbiosis and its role in pediatric disease progression. Given the dynamic nature of the developing microbiome, early and targeted interventions may offer a critical opportunity to prevent disease progression and long-term hepatic complications. Future interventional studies focused on pediatric population are essential to translate microbiome research into precision-based preventive and therapeutic approaches for MASLD in children. Progress in this field will depend on larger, well-characterized longitudinal cohorts and carefully designed randomized trials. Mother–child studies and early-life microbiome tracking may help define developmental risk pathways. Integration of microbiome data with metabolomic and clinical phenotyping may eventually support risk stratification and more individualized care. In conclusion, modulation of the gut microbiota offers a promising approach for preventing and managing pediatric MASLD, but its clinical role is still being defined. Early detection and timely intervention during childhood may help prevent the disease progression reduce the long-term risk of metabolic and liver-related complications.
